# Development of Micro-CT-Based Anatomically Accurate Tooth Model for Finite Element Analysis of Composite Restorations

**DOI:** 10.3390/dj14050279

**Published:** 2026-05-08

**Authors:** Tamás Tarjányi, Balázs Szabó, Lívia Vásárhelyi, Tibor Nagy, Ferenc Farkas, Attila Nagy

**Affiliations:** 1 Department of Medical Physics and Informatics, University of Szeged, Korányi Fasor 9, 6720 Szeged, Hungary; nagy.attila@med.u-szeged.hu; 2 Department of Periodontology, Faculty of Dentistry, University of Szeged, Tisza L. Körút 64-66, 6720 Szeged, Hungary; drszabobalazs77@gmail.com; 3 Interdisciplinary Excellence Centre, Department of Applied and Environmental Chemistry, University of Szeged, Rerrich Béla Tér 1, 6720 Szeged, Hungary; vasarhelyi@chem.u-szeged.hu; 4 ELI ALPS, ELI-HU Non-Profit Ltd., Wolfgang Sandner u. 3, 6728 Szeged, Hungary; nagy.tibor.engineer@gmail.com; 5 Department of Mechanical Engineering, Faculty of Engineering, University of Szeged, Mars Tér. 7, 6724 Szeged, Hungary; farkasf@mk.u-szeged.hu

**Keywords:** CT scan, finite element analysis, 3D CAD, simulation, teeth, mechanical stress

## Abstract

Background: Finite element analysis (FEA) has become an important tool in restorative dentistry for investigating stress distribution in teeth and dental restorations. However, the accuracy of such analyses strongly depends on the anatomical fidelity of the underlying tooth models, which is often limited in simplified geometries. The objective of this study was to develop an anatomically accurate three-dimensional tooth model based on micro-computed tomography (micro-CT) data and to evaluate the biomechanical behaviour of sound and composite-restored teeth under clinically relevant loading conditions. Methods: A human tooth was scanned using high-resolution micro-CT imaging. Enamel, dentin, and pulp were segmented and reconstructed into three-dimensional geometries, which were further refined using computer-aided design (CAD) tools. The resulting models were imported into a finite element environment for mechanical simulation. Static loading conditions were applied to both sound and composite-restored tooth models, including a vertical load of 200 N and an oblique load of 200 N applied at a 45° angle to the tooth crown. Von Mises stress distributions were evaluated to characterize stress concentration patterns. Results: Finite element simulations revealed maximum von Mises stresses of approximately 140 MPa, predominantly localized in the coronal regions of the tooth. Oblique loading produced increased and more asymmetric stress concentrations than vertical loading, particularly in the anterior and posterior crown regions. While overall stress distributions were comparable between sound and composite-restored teeth, locally increased stress levels were observed in restored models under oblique loading. Conclusions: Anatomically accurate, micro-CT-based finite element tooth models provide a robust framework for biomechanical analysis in restorative dentistry. The presented workflow enables detailed evaluation of stress distribution in composite-restored teeth and may contribute to improved understanding and optimization of restorative materials and treatment strategies.

## 1. Introduction

Composite resin restorations are widely used in contemporary restorative dentistry due to their favourable aesthetic properties and continuously improving mechanical performance. Over the past decades, significant progress has been achieved in optimizing the mechanical characteristics of dental composites, including elastic modulus, fracture resistance, fatigue behaviour, and wear resistance. These properties play a critical role in the long-term clinical performance of restorations under functional masticatory loading conditions [1,2,3,4].

Modern dental composites typically consist of a polymer-based resin matrix reinforced with inorganic filler particles. The resin matrix is commonly composed of dimethacrylate monomers such as bisphenol A-glycidyl methacrylate (Bis-GMA) and urethane dimethacrylate (UDMA), which polymerize to form a cross-linked network. Inorganic fillers, often based on glass or ceramic particles, enhance stiffness, strength, and wear resistance [2,3,5]. Additional components, including silane coupling agents and photoinitiators, are incorporated to improve filler-matrix adhesion and enable light-activated polymerization.

Beyond material properties, the biomechanical behaviour of restored teeth is strongly influenced by tooth anatomy, restoration geometry, and loading direction. Experimental investigation of stress distribution within teeth is challenging; therefore, numerical approaches such as finite element analysis (FEA) have become increasingly important in dental biomechanics research [6,7,8,9]. FEA enables detailed analysis of stress and strain distributions in complex three-dimensional tooth structures subjected to simulated occlusal forces, including both vertical and oblique loading scenarios that are representative of clinical mastication [10,11]. The reliability of finite element simulations, however, strongly depends on the anatomical accuracy of the underlying tooth models. Simplified or idealized geometries may fail to capture local stress concentrations, particularly in restored teeth. Micro-computed tomography (micro-CT) provides a non-destructive imaging technique capable of resolving fine anatomical details of dental tissues based on differences in X-ray attenuation [12,13]. Micro-CT data can be segmented into individual tooth components, such as enamel, dentin, and pulp, and subsequently converted into three-dimensional models suitable for finite element simulations [6,14,15,16]. In addition to geometric factors, the biomechanical behaviour of teeth is influenced by several complex variables, including the anisotropic nature of dental tissues, the mechanical behaviour of adhesive interfaces, and variability in clinical conditions such as restoration design and loading history. These factors are often simplified or neglected in numerical models but may significantly affect stress distribution patterns. Beyond mechanical aspects, internal tooth morphology may also influence fluid-dynamic processes relevant to clinical outcomes such as irrigation efficiency in endodontic treatments [17,18].

Several studies have demonstrated the applicability of micro-CT-based finite element modelling in dentistry, including investigations of stress distribution in natural teeth, endodontically treated teeth, and restored structures [6,10,19,20,21,22]. Detailed and reproducible descriptions of the complete workflow from image acquisition and segmentation through geometric refinement and numerical simulation are still limited. Many studies rely on simplified or partially described workflows, and the critical step of transforming voxel-based image data into simulation-ready, anatomically consistent solid models is often insufficiently documented. Moreover, systematic approaches for CAD-based geometric refinement that ensure watertight, mechanically valid models are rarely described in detail.

Therefore, the novelty of the present study lies in the development and explicit documentation of a complete and reproducible workflow, integrating high-resolution micro-CT imaging, segmentation, advanced CAD-based geometric refinement, and finite element analysis. A key contribution is the introduced refinement strategy, which enables the transformation of voxel-derived geometries into smooth, simulation-ready models while preserving anatomical accuracy. Particular emphasis was placed on the influence of loading direction on stress distribution, using clinically relevant vertical and oblique force scenarios.

## 2. Materials and Methods

**Figure 1:**
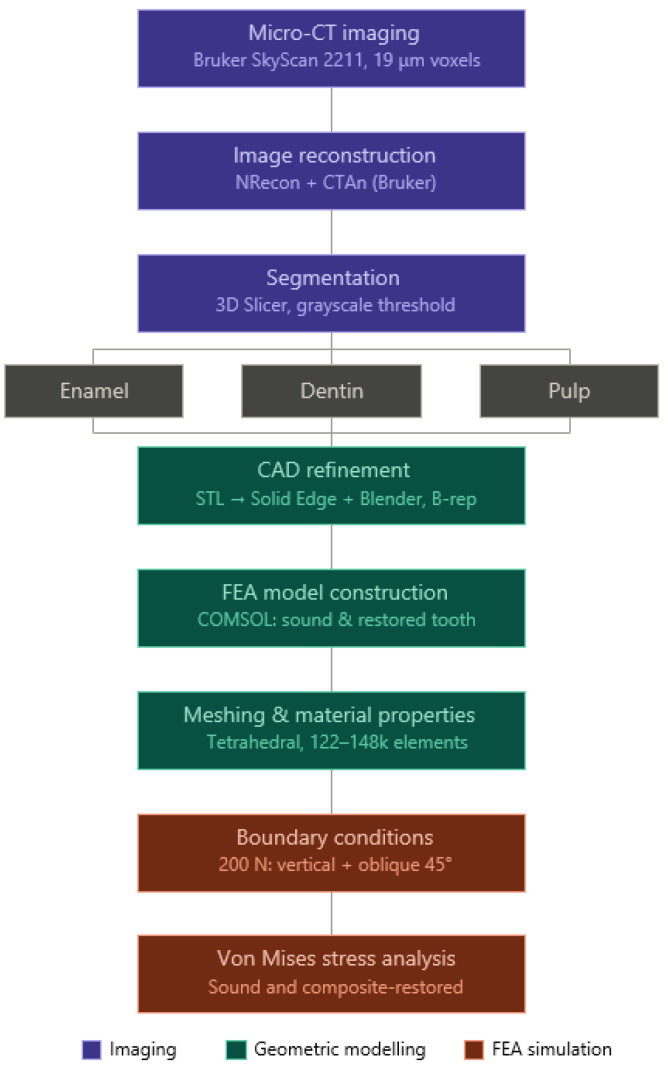
Schematic overview of the workflow for generating anatomically accurate finite element tooth models. The process includes micro-CT imaging, image reconstruction, segmentation of dental tissues (enamel, dentin, pulp), CAD-based geometric refinement, finite element model construction, meshing and material assignment, application of boundary conditions, and von Mises stress analysis.

A schematic overview of the workflow that was applied in the study is provided in Figure 1. The complete pipeline consists of micro-CT image acquisition through segmentation, CAD-based geometric refinement, and finite element analysis. This workflow highlights the key processing steps required to transform voxel-based imaging data into simulation-ready, anatomically accurate models.

### 2.1. Sample Prearation and Micro-CT Scan Protocol

Micro-computed tomography (micro-CT) measurements were performed using a Bruker SkyScan 2211 nanotomograph (Bruker microCT, Kontich, Belgium). An extracted human tooth specimen was used for the study before and after dental filling, the cavity and restoration geometry were designed to reflect a clinically realistic direct composite restoration, while also remaining suitable for segmentation-based modelling and meshing. The intention was to reproduce a clinically plausible geometry that allows meaningful comparison of stress distribution between sound and restored tooth configurations.

The cavity preparation corresponded to an extended Class V–type lesion located on the labial surface, designed to simulate cavity to restore lesions caused by cervical resorption involving both the crown and root. The cavity extended circumferentially over approximately 180° along the mesial–labiar–distal (M–L–D) surfaces. In the apico-coronal direction, the cavity height was 2.5–3.0 mm. The cavity depth was defined relative to the mesio-distal (MD) width of the tooth and set to approximately 40% of the MD dimension.

The sample was mounted on a copper rotating sample holder using dental wax to ensure mechanical stability during scanning. The sample was rotated over 180° with a rotation step of 0.1°, resulting in a total of 2084 projection images acquired at an isotropic voxel size of 19 μm. An open-type, pumped X-ray source was operated at a tube voltage of 120 kV and an emission current of 100 μA. Image acquisition was performed using a 3-megapixel cooled flat-panel detector with an exposure time of 37 ms per projection. To reduce beam-hardening artifacts, a 0.5 mm titanium filter was positioned between the X-ray source and the sample [12,13,23].

**Figure 2:**
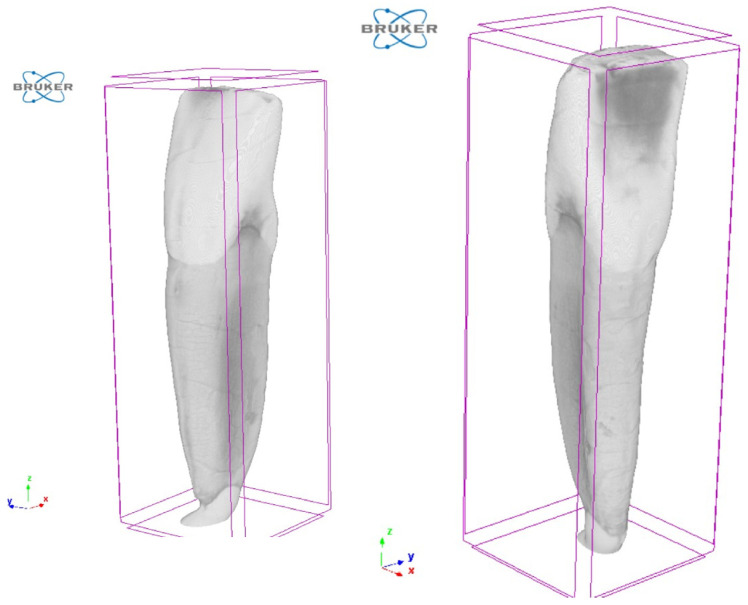
Micro-computed tomography (micro-CT) images of the tooth specimen acquired using a Bruker SkyScan system and visualized in the manufacturer’s reconstruction software (NRecon/CTAn). The images illustrate the raw scan data used for subsequent segmentation of enamel, dentin, and pulp structures.

Reconstruction of the projection images was carried out using NRecon software (version 1.7.1.6, Bruker microCT, Kontich, Belgium), applying standard correction algorithms for misalignment, beam hardening, and ring artifacts. The reconstructed image stack was further processed using CTAn software (version 1.20, Bruker microCT, Kontich, Belgium), see Figure 2. Segmentation was performed based on grayscale intensity differences corresponding to enamel, dentin, and pulp tissues. A median filter was applied to reduce image noise, and isolated objects smaller than 5 × 5 × 5 voxels were removed to eliminate reconstruction artifacts. Three-dimensional volume renderings were generated using CT-Vox software (version 3.3 r1403, Bruker microCT, Kontich, Belgium) for visual inspection and quality control [24,25].

### 2.2. Three-Dimensional Tooth Model

**Figure 3:**
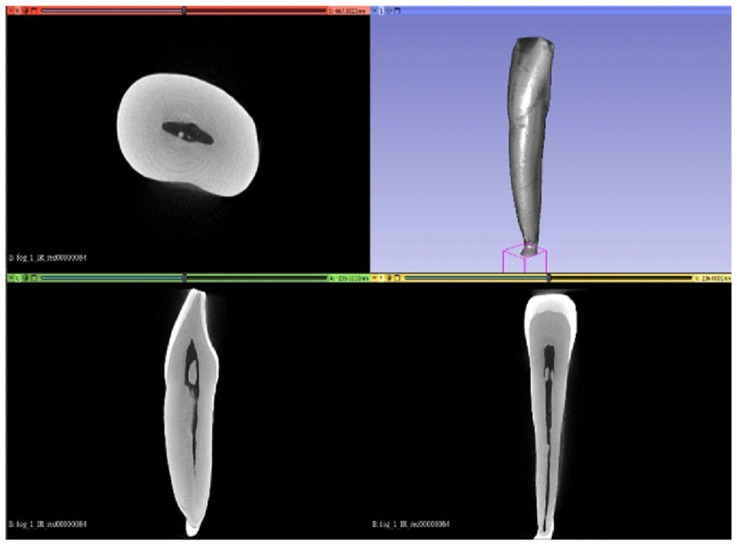
Segmentation of enamel, dentin, and pulp tissues in 3D Slicer based on micro-CT images, shown in axial, coronal, sagittal, and three-dimensional views.

The generation of three-dimensional tooth models was performed through a multi-step workflow involving segmentation, surface processing, and geometric refinement. The reconstructed micro-CT image stack was imported into the 3D Slicer software (v.5.1), where segmentation of the enamel, dentin, and pulp tissues was carried out based on differences in X-ray attenuation, see Figure 3 [26]. Each segmented anatomical structure was treated as a separate segment to allow independent processing and modelling.

**Figure 4:**
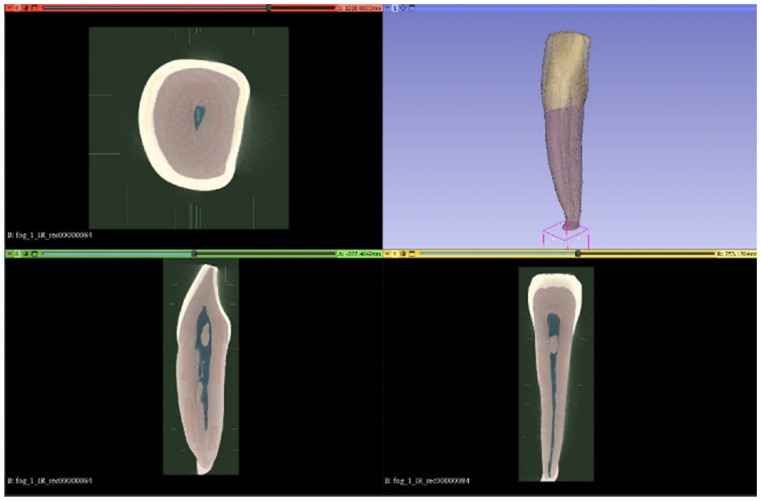
Segmented tooth components displayed as individual segments in 3D Slicer (enamel, dentin/root), visualized using colour coding.

Following segmentation, the individual segments were converted into surface mesh models and exported in STL format, see Figure 4. To improve surface quality, smoothing operations were applied, and minor artifacts originating from the reconstruction process were corrected. These STL files were subsequently imported into Solid Edge CAD software (2023, Siemens, Osaka, Japan) using the “heal mesh faults” option to correct topological inconsistencies and prepare the meshes for further geometric manipulation. Additional surface modifications were performed when required to restore anatomical continuity, such as compensating for enamel abrasion or surface irregularities introduced during sample preparation. In the present study, selected surface corrections were carried out using Blender software (v.3.8) by locally thickening the outer enamel surface. Alternative mesh-editing tools, such as Meshmixer or ZBrush, may also be suitable for similar modifications. After surface corrections, polygon count reduction was applied to optimize computational efficiency while preserving anatomical accuracy [27,28].

**Figure 5:**
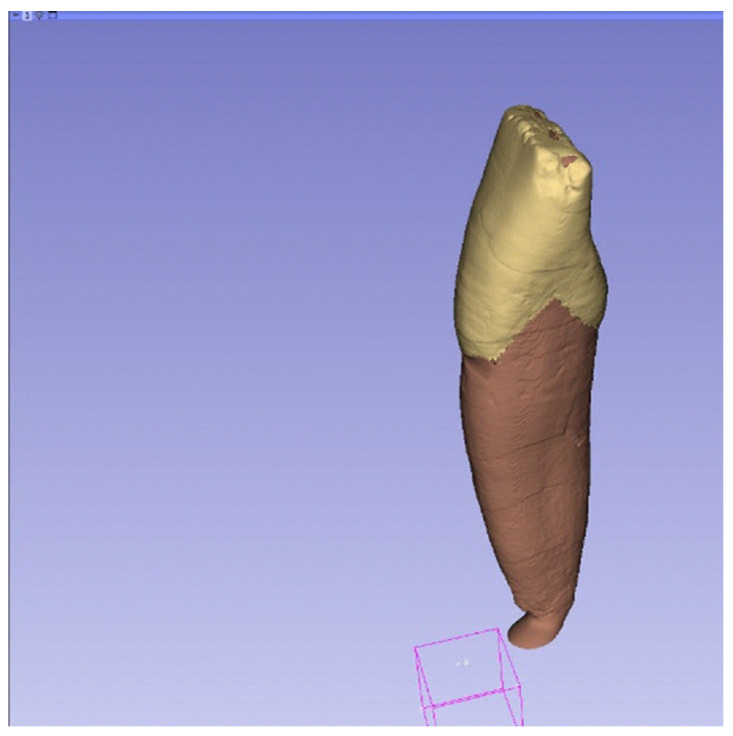
Final three-dimensional tooth model following segmentation and surface processing, comprising enamel, dentin, and pulp components.

The finalized enamel, dentin, and pulp surface models were then transferred to the CAD environment for further parameterization and preparation for finite element analysis. Surface continuity and smooth transitions between anatomical components were ensured to facilitate reliable volume generation and subsequent numerical simulation, see Figure 5 [6,19].

### 2.3. Numerical Simulation Protocol

**Figure 6:**
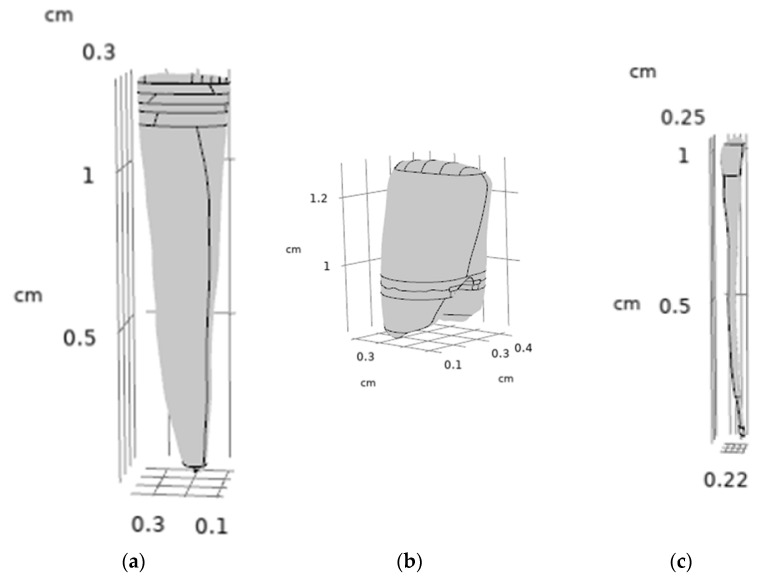

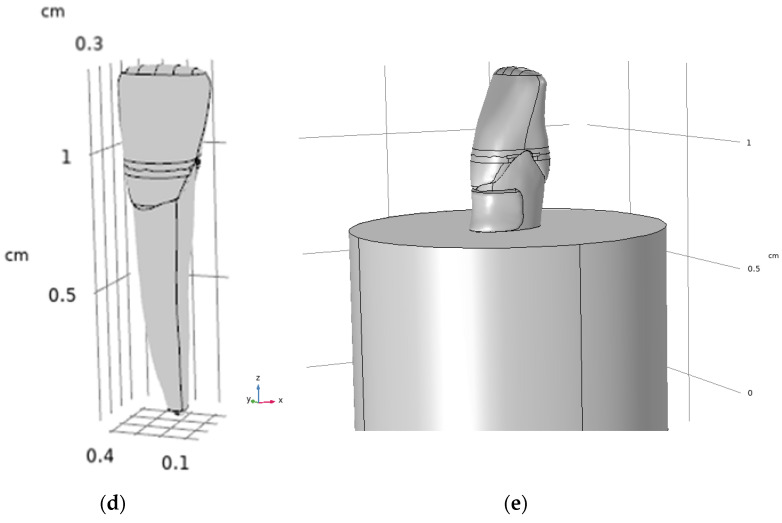
Geometry preparation workflow in the finite element environment. The individual anatomical components are shown as imported into COMSOL Multiphysics: (**a**) dentin, (**b**) enamel, and (**c**) pulp. These components were combined using Boolean operations to form the complete tooth structure (**d**), followed by the addition of the embedding resin and composite restoration to obtain the final simulation geometry (**e**).

Finite element simulations were performed using the anatomically reconstructed three-dimensional tooth models derived from the micro-CT data. Following geometric refinement in the CAD environment, the enamel, dentin, and pulp components were imported individually into COMSOL Multiphysics software (version 5.5, COMSOL Inc., Burlington, MA, USA) for mechanical analysis. The solid mechanics module was employed, and all simulations were conducted using a stationary study to evaluate stress distributions under static loading conditions. Within the simulation environment, the dentin and enamel components were first combined using a union operation to form a continuous tooth structure. Subsequently, the pulp volume was subtracted from the combined geometry to generate the internal pulp chamber. To replicate experimental embedding conditions commonly used in mechanical testing, an acrylic embedding resin was modelled as a cylindrical volume surrounding the tooth. The embedding cylinder had a radius of 0.65 cm and a height of 0.85 cm, with its upper boundary aligned with the lower surface of the composite restoration, see Figure 6 [6,7,10].

Material properties were assigned to each component based on values reported in the literature. All materials were assumed to be homogeneous, isotropic, and linearly elastic. The elastic modulus, Poisson’s ratio, and density values used for enamel, dentin, dental composite, and acrylic embedding resin are summarized in Table 1 [2,3,29].

Two loading scenarios were investigated to represent clinically relevant masticatory conditions. A load magnitude of 200 N was selected to represent a clinically plausible bite force for an anterior tooth under incisal loading conditions. The purpose of this choice was to compare stress patterns under a moderate static load applied in axial and oblique directions, rather than to reproduce the full range of patient-specific masticatory forces. In the first case, a simple vertical static load of 200 N was applied to a defined area on the occlusal surface of the enamel, oriented parallel to the longitudinal (z) axis of the tooth. In the second case, a static load of 200 N was applied at a 45° angle in the x–z plane to simulate oblique loading conditions encountered during mastication. In both cases, the lower surface of the embedding resin was defined as a fixed boundary condition. Meshing was performed using the predefined normal meshing setting in COMSOL. The resulting tetrahedral meshes consisted of element sizes ranging from 0.027 cm to 0.15 cm. Separate meshes were generated for the sound and composite-restored tooth models to ensure accurate geometric representation. Von Mises stress distributions were calculated to evaluate stress concentration patterns within the tooth structures and restorative materials [11,30,31]. To ensure that the numerical results were independent of mesh resolution, a mesh convergence analysis was performed. Three mesh densities (coarse and finer) were generated, and the resulting maximum von Mises stress values were compared. The difference in peak stress between the medium mesh (122,746 elements) and the finer mesh was below 1%, indicating convergence of the solution. Therefore, the fine mesh was considered sufficient to provide an optimal balance between computational efficiency and numerical accuracy, and was used for all subsequent simulations.

## 3. Results

### 3.1. CAD-Based Geometric Refinement of the Tooth Model

Following surface mesh generation from the segmented micro-CT data, further geometric refinement was required to obtain simulation-ready solid models. The STL representations of enamel, dentin, and pulp exhibited locally irregular triangular surfaces.

**Figure 7:**
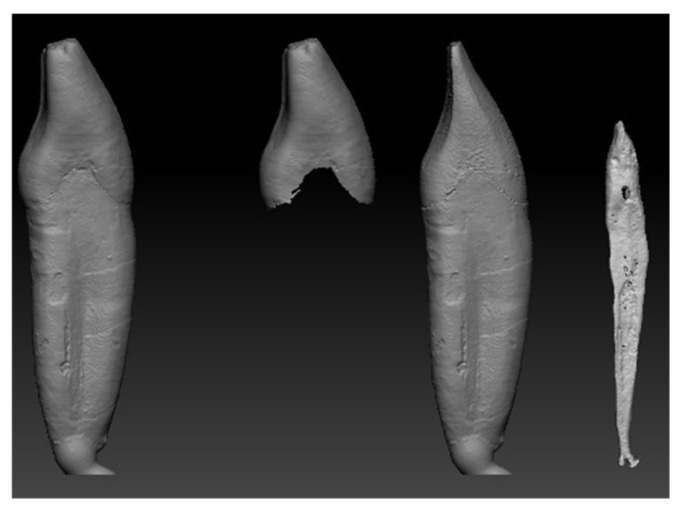
Thickness correction of the segmented tooth components in the ZBrush. Local surface editing was applied to the enamel and dentin to restore anatomical continuity and ensure suitable wall thickness for subsequent solid modelling and finite element simulation.

**Figure 8:**
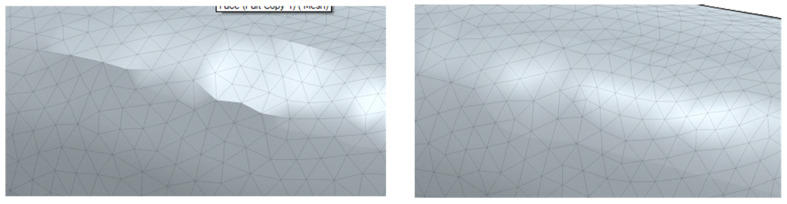
Surface mesh refinement of the pulp geometry during CAD-based preprocessing: (**a**) original STL surface derived from micro-CT segmentation and (**b**) refined surface after mesh smoothing to reduce triangulation artifacts and improve surface continuity.

To Improve surface quality while preserving the original volume, the geometries were remeshed and smoothed in Zbrush (version 2023.2.2, Maxon Computer GmbH, Bad Homburg vor der Höhe, Germany). In addition, local thickness corrections were applied to the enamel and dentin to compensate for reconstruction artifacts and surface abrasion, see Figure 7 and Figure 8.

**Figure 9:**
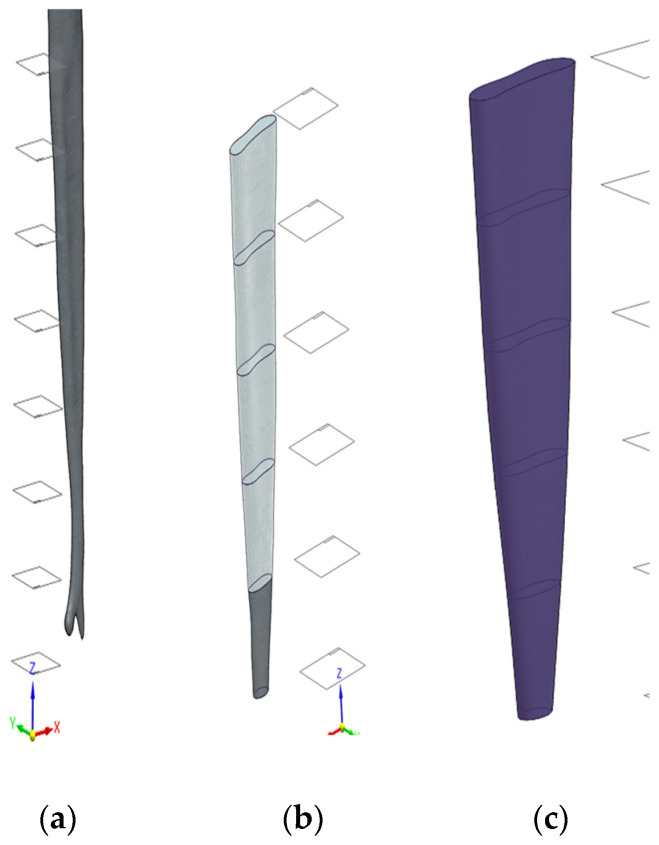
Slice-based CAD reconstruction of the pulp geometry from the triangulated STL model. (**a**) Generation of parallel reference planes along the longitudinal (z) axis and subdivision of the original STL geometry into multiple sections using multi-body and split operations. (**b**) Creation of spline curves on each cross-sectional plane to approximate the anatomical contours of the pulp cavity. (**c**) Reconstruction of smooth B-rep surfaces (blue) using surface interpolation (“BlueSurf”) based on the spline curves, replacing the irregular triangulated STL surface with a continuous representation.

**Figure 10:**
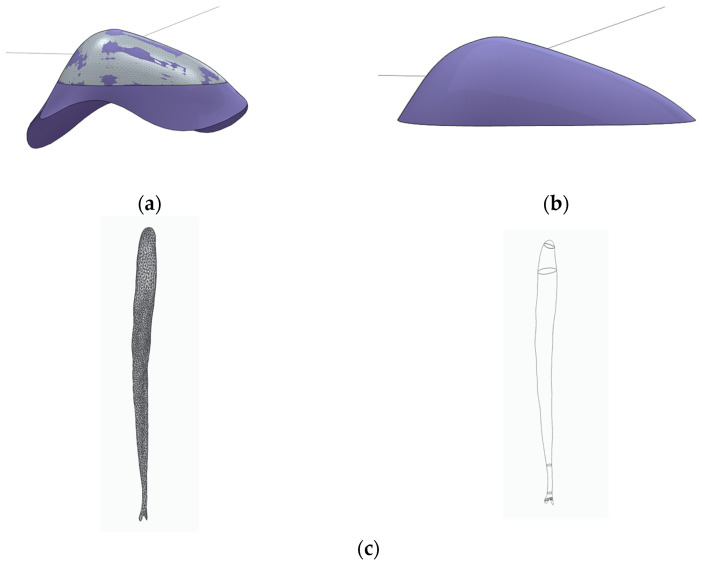
Surface reconstruction of the pulp chamber geometry: (**a**) original triangulated STL surface with fitted B-spline patches, (**b**) resulting smooth B-rep model after surface reconstruction, and (**c**) comparison of the original STL pulp model (**left**) and the reconstructed B-rep body model (**right**). The reconstructed surface set forms a closed volume, enabling generation of a solid model suitable for subsequent Boolean operations and finite element meshing.

**Figure 11:**
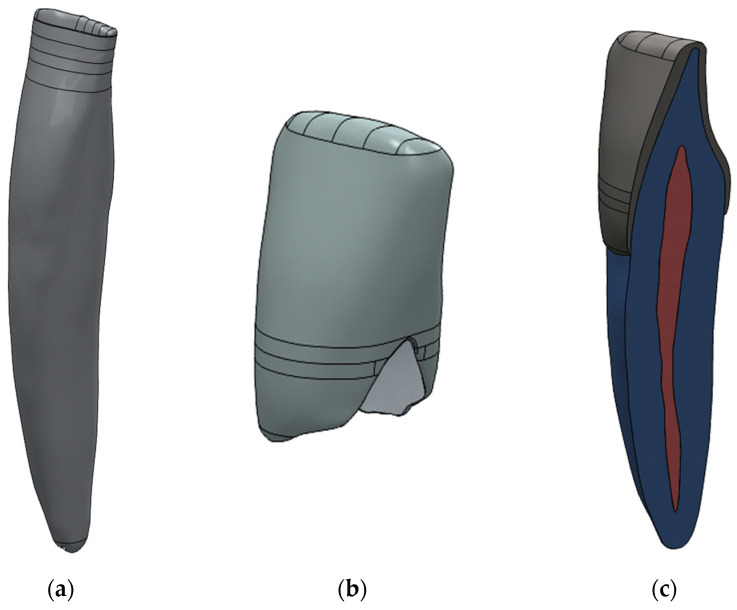
Final CAD-refined tooth components: (**a**) dentin, (**b**) enamel, and (**c**) assembled three-dimensional tooth model after geometric refinement. The geometries represent smooth, watertight B-rep solids suitable for finite element modelling.

The original triangular mesh was replaced by a smooth, continuous surface representation by removing mesh vertices and reconstructing the geometry using fitted spline surfaces in CAD environment. To facilitate controlled remodelling, a series of parallel planes were generated along the longitudinal axis of the pulp chamber. These planes were used to divide the geometry into multiple cross-sectional segments using multi-body and split operations. For each intersecting plane, spline-based sketches were created to capture the local anatomical contours. Using these sketches, B-rep surface models were generated via surface interpolation commands, resulting in a continuous, anatomically consistent surface representation. The individual surface patches were merged using surface stitching operations with a defined geometric healing, producing a closed volumetric model B-rep body model. This yielded a watertight solid representation suitable for Boolean operations and finite element meshing. A similar refinement strategy was applied to the dentin and enamel components, with minor modifications to account for differences in geometry and thickness. The final tooth components were assembled by coordinate matching to ensure precise spatial alignment. The CAD-based refinement process enabled the transformation of voxel-derived surface meshes into smooth, watertight solid models, which are essential for robust finite element simulations. The main steps of the CAD editing workflow are illustrated in Figure 9, Figure 10 and Figure 11.

### 3.2. Geometry Preparation and Finite Element Model Construction

The segmented, CAD-refined enamel, dentin, and pulp surface models were imported into the finite element environment and assembled into a continuous three-dimensional tooth geometry. Boolean union operations were used to combine the enamel and dentin volumes, while the pulp chamber was generated by subtracting the pulp volume from the combined structure.

An acrylic embedding resin was added as a surrounding cylindrical volume to replicate experimental fixation conditions commonly used in mechanical testing. The final simulation geometries were generated for both the sound tooth and the composite-restored tooth models. In the restored configuration, the composite filling was integrated into the coronal region, ensuring proper contact with the surrounding dental tissues. The geometry preparation workflow is illustrated in Figure 6.

### 3.3. Mesh Generation

**Figure 12:**
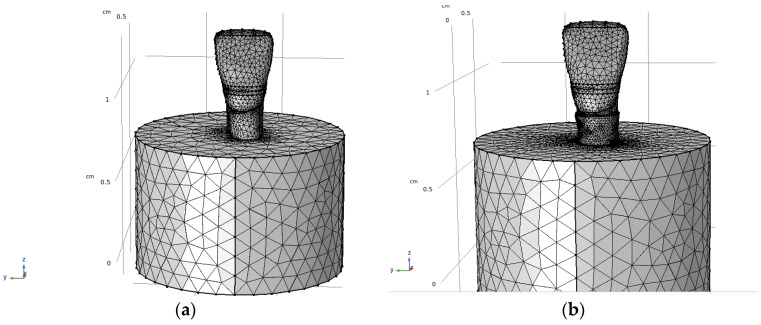
Finite element meshes of the three-dimensional tooth models embedded in acrylic resin: (**a**) sound tooth model and (**b**) composite-restored tooth model.

Finite element meshes were generated for both the sound and composite-restored tooth models using tetrahedral elements. The meshing strategy provided sufficient resolution to accurately represent the complex tooth geometry while maintaining computational efficiency. Element sizes ranged from 0.027 cm to 0.15 cm. The sound tooth model consisted of 122,746 tetrahedral elements, including 17,426 triangular surface elements and 2019 edge elements. The composite-restored tooth model contained 147,868 tetrahedral elements, with 23,384 triangular surface elements and 2502 edge elements. The resulting meshes are shown in Figure 12.

### 3.4. Loading Configurations

**Figure 13:**
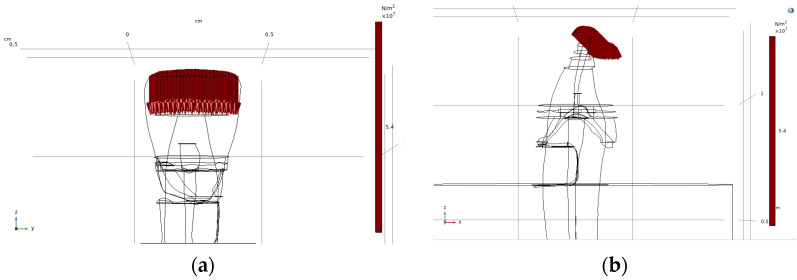
Loading configurations applied in the finite element simulations: (**a**) vertical loading parallel to the longitudinal axis of the tooth and (**b**) oblique loading applied at a 45° angle in the x–z plane.

Two static loading configurations were applied to evaluate stress distribution under clinically relevant masticatory conditions. In the first configuration, a vertical load of 200 N was applied to the occlusal surface of the tooth crown, oriented parallel to the longitudinal axis of the tooth. In the second configuration, a load of 200 N was applied at a 45° angle in the x–z plane to simulate oblique loading conditions. The applied force directions and loading locations are illustrated in Figure 13.

### 3.5. Stress Distribution in the Sound Tooth Model

Under vertical loading, the sound tooth model exhibited maximum von Mises stresses of approximately 140 MPa, predominantly localized in the coronal region above the level of the embedding resin. Increased stress concentrations were observed mainly on the lingual aspect of the crown, while stress levels decreased progressively toward the apical region of the root. Minimal stress was detected in the anterior region of the tooth.

**Figure 14:**
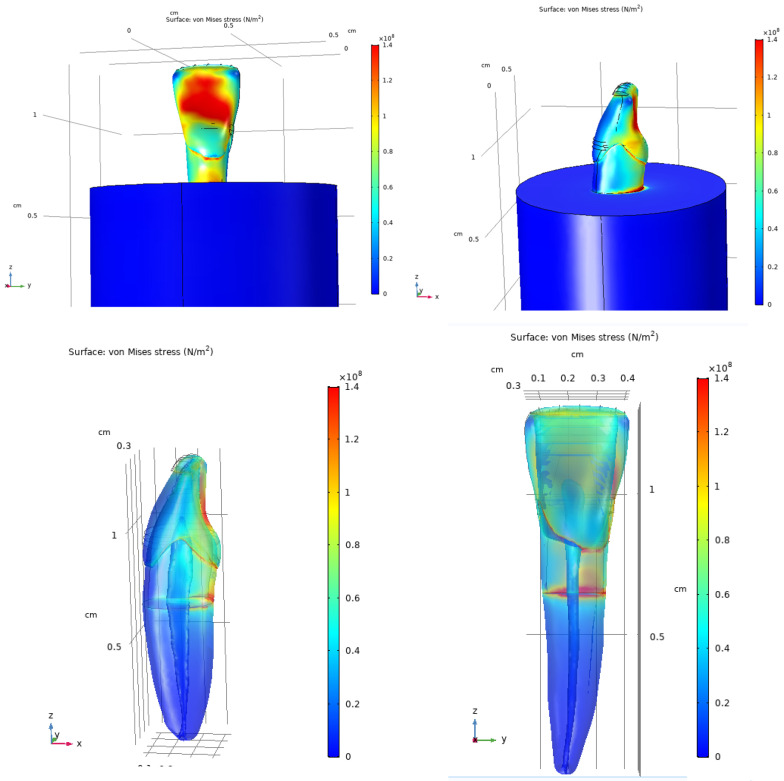
Von Mises stress distribution in the sound tooth model under vertical loading (200 N), shown from two viewing angles and a transparent view.

**Figure 15:**
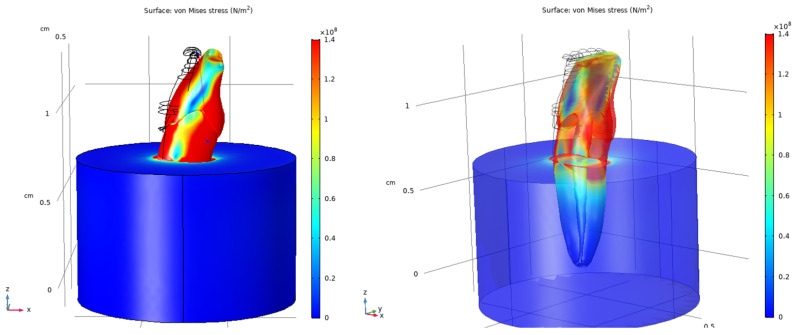
Von Mises stress distribution in the sound tooth model under oblique loading (200 N at 45°), shown in opaque and transparent views to visualize the pulp chamber.

Under oblique loading, increased and more asymmetric stress distributions were observed compared with vertical loading. Stress concentrations were primarily localized in the anterior and posterior regions of the crown, whereas reduced stress levels were observed on the lateral surfaces. The von Mises stress distributions for both loading conditions are shown in Figure 14 and Figure 15.

### 3.6. Stress Distribution in the Composite-Restored Tooth Model

The composite-restored tooth model exhibited stress distribution patterns similar to those observed in the sound tooth under vertical loading. Maximum von Mises stresses of approximately 140 MPa were localized in the coronal region near the occlusal surface. Increased stress levels were observed in proximity to the restoration margins, although the overall stress distribution remained comparable to that of the sound tooth.

**Figure 16:**
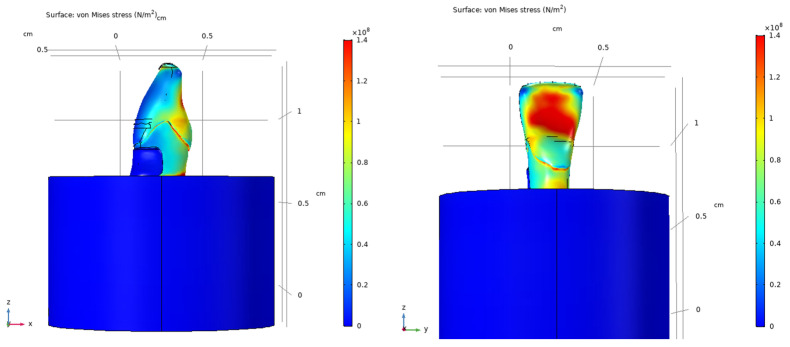
Von Mises stress distribution in the composite-restored tooth model under vertical loading (200 N), shown from two viewing angles.

**Figure 17:**
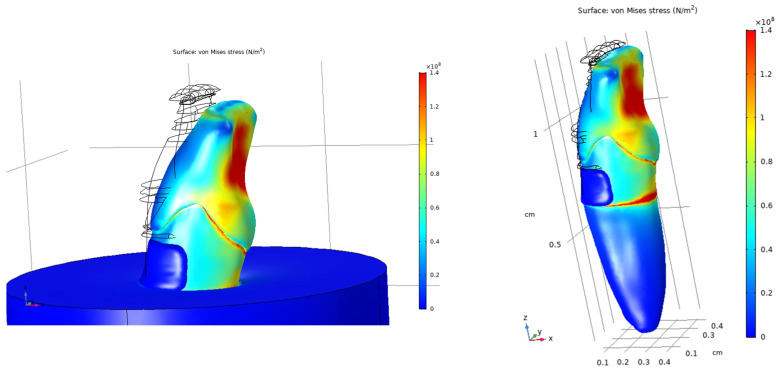
Von Mises stress distribution in the composite-restored tooth model under vertical loading (200 N), shown from viewing angles.

**Figure 18:**
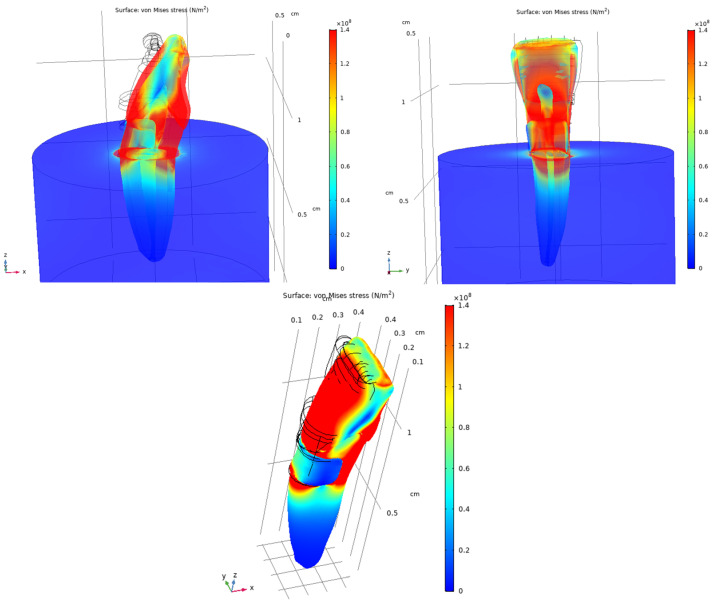
Von Mises stress distribution in the composite-restored tooth model under oblique loading (200 N at 45°), shown in different views.

Under oblique loading, the composite-restored model showed more pronounced stress concentrations than those observed under vertical loading. Local increases in von Mises stress were evident around the composite restoration and adjacent dental tissues. The stress distributions for the restored tooth model are presented in Figure 16, Figure 17 and Figure 18.

### 3.7. Line-Based Stress Analysis

**Figure 19:**
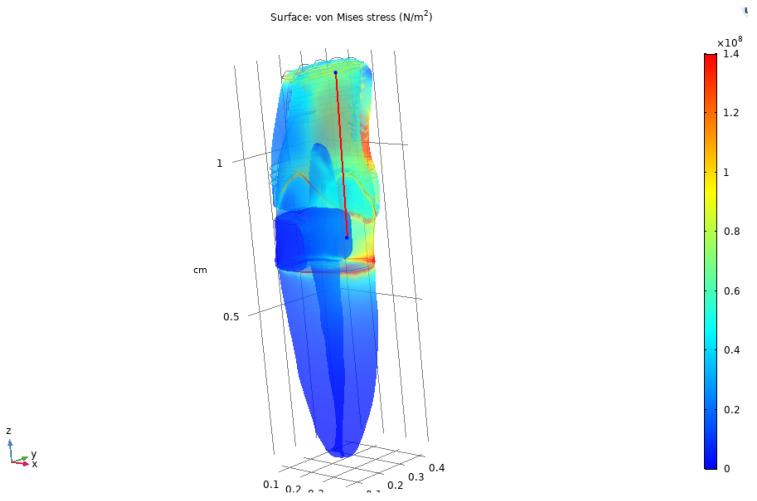
A line was selected along the tooth model to analyse the stress distribution.

To further quantify stress variation along the tooth structure, a linear path was defined from the coronal region toward the root along the longitudinal axis of the tooth, see Figure 19. Von Mises stress values were extracted along this line for both sound and composite-restored tooth models.

**Figure 20:**
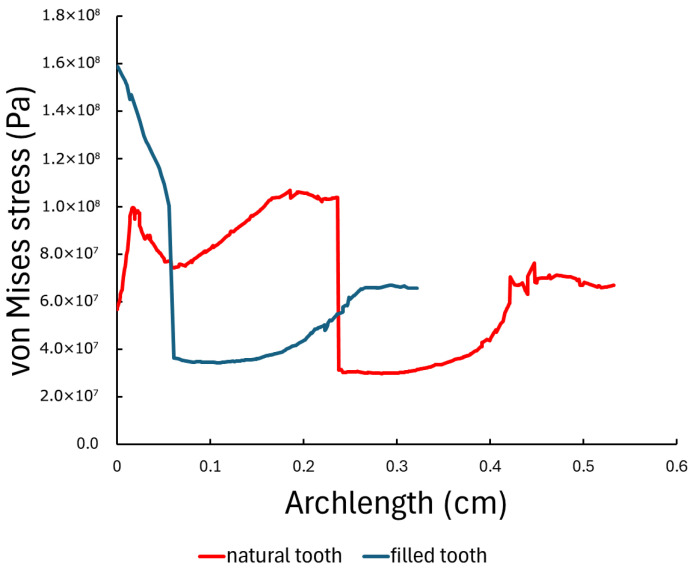
Von Mises stress distribution along the selected longitudinal line in the sound and composite-restored tooth models under vertical loading (200 N).

**Figure 21:**
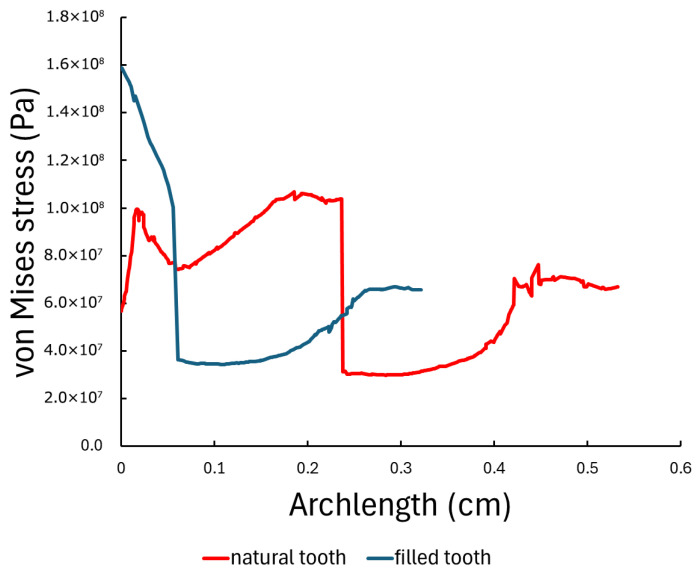
Von Mises stress distribution along the selected longitudinal line in the sound and composite-restored tooth models under oblique loading (200 N at 45°).

Under vertical loading, the sound tooth model exhibited a peak stress of approximately 110 MPa near the enamel surface, followed by a gradual decrease toward the apical region. In contrast, the composite-restored tooth showed an increased initial peak stress of approximately 160 MPa, with a more rapid reduction along the path length. Under oblique loading, both models exhibited substantially increased peak stresses, reaching approximately 300 MPa, with similar stress distribution profiles along the selected path. The extracted stress profiles are shown in Figure 20 and Figure 21.

## 4. Discussion

### 4.1. Summary of the Present Results

Finite element analysis has become an increasingly important tool in restorative dentistry for evaluating stress distribution within teeth and dental restorations under simulated functional loading. The present study demonstrates a reproducible workflow for generating anatomically accurate, micro-CT–based tooth models and applying finite element analysis to investigate the biomechanical behaviour of sound and composite-restored teeth. A particular strength of the proposed approach lies in the integration of high-resolution imaging, CAD-based geometric refinement, and clinically relevant loading scenarios.

One of the main challenges in finite element modelling of teeth is the transformation of voxel-based micro-CT data into smooth, watertight solid geometries suitable for numerical simulation. In the present study, this challenge was addressed through a CAD-based refinement strategy involving surface smoothing, thickness correction, and spline-based surface reconstruction. These steps were essential for eliminating reconstruction-related surface irregularities and ensuring geometric continuity between anatomical components. Similar difficulties have been reported in previous micro-CT–based dental FEA studies, although detailed descriptions of the geometric refinement process are often limited or omitted [6,7,10]. By explicitly documenting this workflow, the present study contributes to improved reproducibility and transparency in dental finite element modelling.

The stress distribution patterns observed in the simulations highlight the importance of loading direction in biomechanical assessments of teeth. Under vertical loading, von Mises stresses were predominantly localized in the coronal region, with stress levels decreasing toward the root. In contrast, oblique loading resulted in increased and more asymmetric stress concentrations, particularly in the anterior and posterior regions of the crown. These findings are consistent with previous finite element investigations demonstrating that non-axial loading conditions generate increased stress levels and may contribute to increased risk of restoration failure or tooth fracture [11,30,31].

Comparison between sound and composite-restored tooth models revealed broadly similar global stress distribution patterns under vertical loading, suggesting that the presence of a composite restoration does not fundamentally alter overall load transfer in the tooth. However, under oblique loading, locally increased stress concentrations were observed in the restored model, particularly near the restoration margins. This observation aligns with earlier studies reporting that restorative interfaces are susceptible to stress amplification under non-axial forces, which may be clinically relevant in the context of marginal degradation and fracture initiation [7,11,32].

The line-based stress analysis further emphasized differences between sound and restored teeth. The composite-restored model exhibited increased peak stresses near the enamel surface under vertical loading, while both models showed substantially increased peak stresses under oblique loading. These results underscore the potential biomechanical significance of oblique masticatory forces, which are frequently encountered during normal function and parafunctional activities. Although the increased local stress peaks observed under oblique loading may indicate mechanically vulnerable regions, these values should not be interpreted directly as clinical fracture sites without experimental validation and more advanced material modelling. Consequently, finite element models that consider only vertical loading may underestimate clinically relevant stress concentrations.

### 4.2. Comparing with Previous Results

Recent finite element studies have emphasized the importance of cavity geometry and material selection in determining stress distribution within restored teeth. Xu et al. employed three-dimensional finite element analysis to systematically investigate the effects of cavity width, depth, and length in Class II restorations, demonstrating that stress distribution follows a nonlinear relationship with cavity dimensions and that intermediate cavity sizes often provide more favourable biomechanical behaviour [33]. Their findings support the concept that neither excessively conservative nor overly extensive cavity preparations are universally optimal, but rather that cavity design should be adapted to the specific clinical situation.

Complementary insights were provided by a recent study in the *Journal of Functional Biomaterials*, which highlighted the critical role of material stiffness and elastic modulus mismatch at dental tissue interfaces in governing stress transfer and localization [34]. That work demonstrated that restorative materials with higher elastic moduli can effectively reduce stress in enamel while potentially increasing stress concentrations in dentin, underscoring the importance of directing stresses toward restorative materials rather than vital tooth structures.

The present study aligns with and extends these findings by integrating anatomically accurate, micro-CT–derived tooth models with CAD-based geometric refinement and clinically relevant loading conditions. While Xu et al. focused primarily on cavity dimension optimization under vertical loading, the inclusion of oblique loading in the current work revealed substantially altered stress distributions, particularly near restoration margins and in coronal regions. This suggests that loading direction represents an additional critical factor that should be considered alongside cavity geometry and material properties when assessing biomechanical performance. Together, these studies reinforce the need for a balanced, patient-specific approach to cavity preparation that accounts for geometry, material behaviour, and functional loading conditions.

### 4.3. Limitations

Several limitations of the present study should be acknowledged. The present study was based on a single extracted tooth; therefore, the numerical results cannot be considered statistically representative of the full population of anterior teeth. However, the primary objective of this work was not to establish population-level biomechanical values, but to develop and document a reproducible image-based workflow that links high-resolution micro-CT imaging, anatomical segmentation, CAD refinement, and finite element simulation. The tooth specimen served as a proof-of-concept model demonstrating that anatomically detailed geometries can be transformed into simulation-ready solid models suitable for stress analysis.

An important limitation of the present model is the omission of the periodontal ligament. In vivo, the periodontal ligament provides compliant support and modifies the transfer and distribution of occlusal forces. Its absence in the current model means that the stress magnitudes and patterns should be interpreted as those of an idealized embedded system rather than as a full physiological representation of tooth support. Incorporation of the periodontal ligament, alveolar bone, and, where feasible, a more detailed cementum layer would further improve clinical realism in future models. The assumption of homogeneous, isotropic, and linearly elastic material behaviour represents another practical simplification. In reality, enamel and dentin are structurally heterogeneous tissues, and their mechanical behaviour depends on microstructure, location, and orientation. The present simulations are best interpreted as comparative biomechanical estimates rather than exact reproductions of tissue-level mechanical behaviour. In addition, the mechanical behaviour of adhesive interfaces, fatigue loading conditions may also influence stress distribution. Incorporation of these aspects may further improve physiological relevance and represents an important direction for future studies.

Although cone-beam CT is more widely available in clinical dentistry than micro-CT, its lower spatial resolution and reduced contrast between hard dental tissues may limit reliable separation of enamel, dentin, and fine internal anatomical details. Therefore, CBCT is not expected to provide the same geometric fidelity as micro-CT for the present type of detailed finite element model. Nevertheless, future work may evaluate whether CBCT-based workflows, potentially combined with automated segmentation methods, could provide clinically useful approximations for selected applications where ultra-high anatomical resolution is not essential. Despite these limitations, such assumptions are commonly employed in dental finite element studies and allow for meaningful comparative analysis of stress distribution patterns [6,10,11].

Future studies should aim to incorporate more detailed representations of supporting tissues, time-dependent material behaviour, and patient-specific loading conditions. Advances in automated and AI-assisted segmentation techniques may further facilitate the generation of anatomically accurate models and reduce the time required for geometric refinement. The present workflow provides a robust foundation for investigating biomechanical aspects of restorative dentistry using finite element analysis.

## 5. Conclusions

This study presents a reproducible workflow for generating anatomically accurate, micro-CT–based tooth models and applying finite element analysis to evaluate the biomechanical behaviour of sound and composite-restored teeth. The integration of high-resolution imaging with CAD-based geometric refinement enabled the creation of simulation-ready models with improved anatomical continuity and numerical stability.

Finite element simulations demonstrated that loading direction has a substantial influence on stress distribution within both sound and restored teeth. While vertical loading resulted in relatively symmetric stress patterns, oblique loading produced increased and more localized stress concentrations, particularly in coronal regions and near restoration margins. These findings highlight the importance of considering non-axial masticatory forces in biomechanical assessments of dental restorations.

The proposed modelling approach provides a robust framework for investigating the effects of geometry, restoration design, and loading conditions on stress distribution in restorative dentistry. It may support future studies aimed at optimizing restorative strategies and improving the long-term biomechanical performance of dental restorations.

## Figures and Tables

**Table 1:** Mechanical parameters used for the numerical calculations in the simulation.

Part	Density [kg/m^3^]	Modulus of Elasticity [GPa]	Poisson’s Ratio
Enamel	2700	84.82	0.3
Dentine	1800	24.35	0.3
Acrylic embedding resin	1200	2	0.2
Dental composite filling	1240	12.5	0.3

## Data Availability

All data generated or analyzed during this study are included in this published article.

## Abbreviations

The following abbreviations are used in this manuscript:

FEA	Finite Element Analysis
micro-CT	Micro-Computed Tomography
CT	Computed Tomography
CAD	Computer-Aided Design
STL	Stereolithography (file format)
B-rep	Boundary Representation
